# Antenatal preventative pelvic floor muscle exercise intervention led by midwives to reduce postnatal urinary incontinence (APPEAL): protocol for a feasibility and pilot cluster randomised controlled trial

**DOI:** 10.1186/s40814-022-01185-y

**Published:** 2022-10-22

**Authors:** D. Bick, J. Bishop, T. Coleman, S. Dean, E. Edwards, H. Frawley, E. Gkini, J. Hay-Smith, K. Hemming, E. Jones, E. Oborn, M. Pearson, V. Salmon, S. Webb, C. MacArthur

**Affiliations:** 1 Warwick Clinical Trials Unit, Warwick Medical School, University of Warwick, Gibbet Hill, Coventry, CV4 7AL UK; 2 University of Birmingham, Birmingham, UK; 3 University of Leicester, Leicester, UK; 4 University of Exeter, Exeter, UK; 5 Birmingham Women’s and Children’s Hospital, Birmingham, UK; 6 University of Melbourne, Melbourne, Australia; 7 University of Otago, Otago, New Zealand; 8 University of Warwick, Coventry, UK; 9 University of Hull, Hull, UK; 10 Royal College of Midwives, London, UK

**Keywords:** Pelvic floor muscle exercises, Pregnancy, Postnatal, Urinary incontinence

## Abstract

**Background:**

Antenatal pelvic floor muscle exercises (PFME) in women without prior urinary incontinence (UI) are effective in reducing postnatal UI; however, UK midwives often do not provide advice and information to women on undertaking PFME, with evidence that among women who do receive advice, many do not perform PFME.

**Methods:**

The primary aim of this feasibility and pilot cluster randomised controlled trial is to provide a potential assessment of the feasibility of undertaking a future definitive trial of a midwifery-led antenatal intervention to support women to perform PFME in pregnancy and reduce UI postnatally.

Community midwifery teams in participating NHS sites comprise trial clusters (*n* = 17). Midwives in teams randomised to the intervention will be trained on how to teach PFME to women and how to support them in undertaking PFME in pregnancy. Women whose community midwifery teams are allocated to control will receive standard antenatal care only.

All pregnant women who give birth over a pre-selected sample month who receive antenatal care from participating community midwifery teams (clusters) will be sent a questionnaire at 10–12 weeks postpartum (around 1400–1500 women). Process evaluation data will include interviews with midwives to assess if the intervention could be implemented as planned. Interviews with women in both trial arms will explore their experiences of support from midwives to perform PFME during pregnancy. Data will be stored securely at the Universities of Birmingham and Exeter.

Results will be disseminated through publications aimed at maternity service users, clinicians, and academics and inform a potential definitive trial of effectiveness. The West Midlands–Edgbaston Research Ethics Committee approved the study protocol.

**Discussion:**

Trial outcomes will determine if criteria to progress to a definitive cluster trial are met. These include women’s questionnaire return rates, prevalence of UI, and other health outcomes as reported by women at 10–12 weeks postpartum. Progress to a definitive trial however is likely to be prevented in the UK context by new perinatal pelvic health service, although this may be possible elsewhere.

**Trial registration:**

10.1186/ISRCTN10833250. Registered 09/03/2020

## Background

Pregnancy and birth are the main risk factors for urinary incontinence (UI) [1]. Prevalence of UI at around 30 weeks gestation has been reported as 31% in nulliparous and 42% in parous women [2]. Postpartum prevalence ranges from 30% in the first 3 months to up to 47% in the first 12 months postpartum [3]. Between two-thirds to three-quarters of women may still experience UI symptoms 12 years after delivery [4] placing a large burden on women’s health and quality of life [5], with pressure on NHS resources and wider societal costs [6].

Despite experiencing UI, many women ‘suffer in silence’ as they are embarrassed or accept symptoms as normal after having a baby, despite evidence that pelvic floor muscle exercises (PFME), if performed correctly in pregnancy, can reduce risk of developing UI after giving birth. A Cochrane review of effectiveness of PFME for prevention and management of urinary and faecal incontinence in pregnant and postnatal populations [7] included 46 trials with a total of 10832 women. Data were analysed according to whether PFME interventions were for prevention of UI (pregnant women without prior UI) or for treatment (pregnant or postnatal women symptomatic of UI) or mixed prevention/treatment trials. In prevention trials, pregnant women without prior UI randomised to PFME teaching and supervision were 29% (RR 0.71,95% CI 0.54 to 0.95) less likely than women randomised to no PFME or usual antenatal care to report UI up to 6 months after giving birth. Mixed prevention/treatment trials were of moderate quality with a possibility of similar reduction in postpartum UI (RR 0.73, 95% 0.55 to 0.97). There was no clear evidence on whether PFME is effective for treating pregnant or postnatal women symptomatic of UI.

Currently most pregnant women without prior UI do not benefit from effective PFME, due to lack of information and support about how to undertake these. Furthermore for antenatal PFME to be effective, teaching should be delivered through a structured training programme (ensuring PFME is performed correctly and regularly) [8] since information provision alone is seldom enough to support long-term (exercise) behaviour change [9].

The Royal College of Midwives and Chartered Society of Physiotherapy published a joint statement recommending that all pregnant women should be offered evidence-based information and advice on PFME [10] but did not address overcoming barriers to implementation on the part of midwives or women [11]. A major policy review by NHS England recognised the importance of maternal perinatal pelvic floor health. The NHS Long-Term plan [12] includes specific recommendations to improve access to postnatal physiotherapy and importance of training and support in pelvic floor health for clinicians, including midwives, working with women. Despite recently updated NICE guidelines for routine antenatal care making no specific mention of PFME in pregnancy [13], guidance does include a link to NICE guidance on pelvic floor dysfunction [14] which recommends that for women using the maternity services, information on preventing pelvic floor dysfunction should be offered at all antenatal and postnatal contacts. The guideline also recommends pelvic floor dysfunction is included as part of the training syllabus for all relevant healthcare professionals [14].

### Feasibility and pilot trial rationale

Translational research focuses on moving discoveries from ‘bench to bedside’ to close the translational gap [15]; however, closing this first translational gap is insufficient to demonstrate full clinical impact of an intervention. In UK maternity services, there is a ‘second translational gap’ between evidence generated and implementation into practice. With a high prevalence of UI following birth, lack of implementation of evidence to prevent UI through use of PFME has a serious cumulative impact on women’s health. Conversations with NHS consultant midwives when developing our study confirmed a lack of consistency in how PFME information was offered in their maternity units, with no formal training to support midwives to deliver structured PFME advice. Of 103 local antenatal women we surveyed to ask about health advice they were offered by their midwives, only 50% could recall any information about PFME and of those who did, half did not perform PFME for a range of reasons. Implementation of PFME would require individual and collective action of women, clinicians, maternity services and organisations, funders and policymakers [16].

The NIHR funded Antenatal Preventative Pelvic Floor Exercises And Localisation (APPEAL) programme includes several linked work packages (WP). The first WP explored perceived organisational, health professional and individual barriers and enablers to implementation of PFME in current UK practice [16]. It involved a critical interpretive synthesis of systematically identified primary quantitative, qualitative studies and research syntheses of women’s and HCPs attitudes, beliefs, or experiences of implementing PFMT [11]. This paper describes the protocol for the feasibility and pilot cluster trial being undertaken as the final WP which is reported in line with the Standard Protocol Items Recommendations for Interventional Trials (SPIRIT) [17].

## Methods and analysis

The primary aim of this feasibility and pilot cluster randomised controlled trial is to assess the feasibility of undertaking a future definitive trial of a midwifery-led antenatal intervention to support women to perform PFME in pregnancy to reduce UI postnatally and assess intervention acceptability to midwives and the women they support.

Specific objectives include feasibility of undertaking a future definitive trial, informed by return rates of questionnaires sent to all women who give birth over a pre-selected 1-month period when they are 10–12 weeks postpartum, prevalence of UI and other health outcomes and feasibility of implementing the trial intervention by midwives at routine antenatal care contacts (Table 1).

**Table 1:** Feasibility and pilot trial objectives

• Provide training for community midwife teams randomised to the intervention arm to encourage the incorporation of a PFME care package into their usual antenatal care	
• Assess if training, intervention implementation and trial processes are acceptable to midwives	
• Assess if midwife characteristics (e.g. years qualified) are similar across trial arms for feasibility	
• Assess questionnaire return rates from women at 10–12 weeks postpartum overall and in both trial arms to inform feasibility and estimate sample size for a full-scale RCT	
**•** Assess characteristics of women overall and within trial arms who return questionnaires compared with all those who gave birth in the same midwifery teams over the same study period but did not respond, using anonymised routine data	
• Assess if baseline characteristics collected following birth (self-reported UI at pregnancy commencement, maternal and obstetric characteristics collated from antenatal booking, labour and birth data) are similar across trial arms to inform feasibility	
• Assess women’s practice of PFME before, during and after pregnancy using women’s postnatal questionnaire and interview data	
• Assess prevalence of UI and faecal incontinence at 10–12 weeks postpartum using women’s questionnaire data to inform the sample size calculation for a full-scale RCT	
• Assess midwife support for PFME in both trial groups using qualitative interviews with midwives, women, and women’s questionnaire data to inform feasibility	
• Undertake any necessary revisions to the APPEAL training package and following this, recommend roll-out by midwives to all pregnant women as part of the NHS Long-Term Plan	

**Fig. 1:**
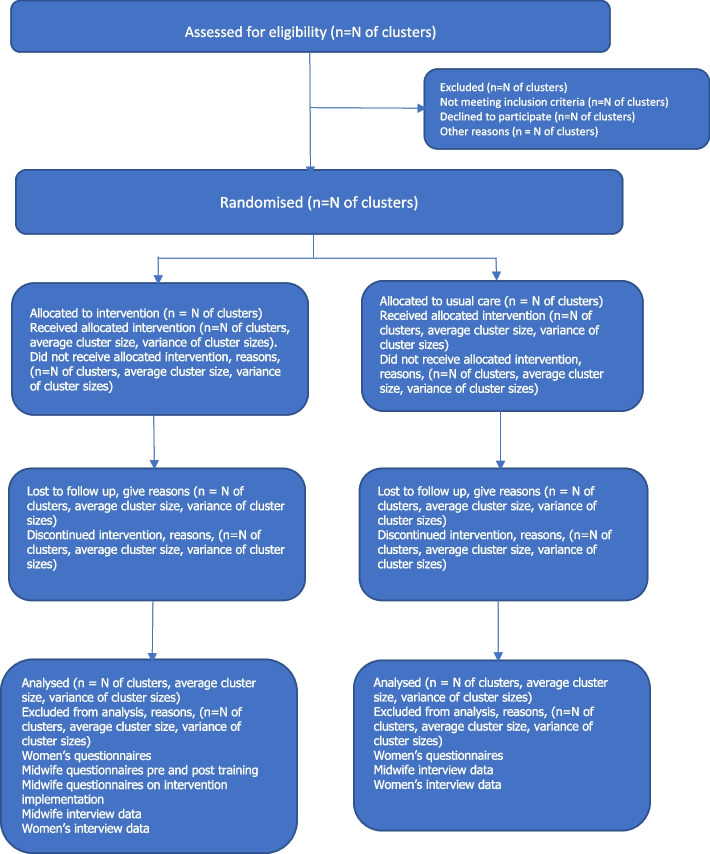
APPEAL Trial Schemata

The trial schematic is shown in Fig. 1.

### Intervention

Intervention components and materials to support behaviour change are underpinned by the Behaviour Change Wheel theoretical framework and the Behaviour Change Technique Taxonomy (version 1) [18, 19] in line with the Medical Research Council’s guidance for developing and evaluating complex interventions (https://www.bmj.com/content/374/bmj.n2061).

Due to strict COVID-19 policies at NHS sites, midwife training will be provided via online interactive video-conferencing with small groups of midwives. Two research midwives will lead and facilitate initial training sessions lasting approximately 2 h. Training handbooks will be sent to intervention cluster midwives before their training. The training package will include an interactive session with vignettes and role-play to enable midwives to raise and discuss the importance of a healthy pelvic floor, benefits of performance of PFME to prevent UI, and a video of how to teach women about PFME. Information is presented on pelvic floor muscle anatomy and function; muscle exercise training principles (physiology), a stepped approach to assessing and teaching the correct muscle contraction, setting an individualised PFME training programme, information on how to screen for serious incontinence problems and criteria for referral.

Following training, midwives will initially introduce the topic of pelvic floor health at the antenatal ‘booking’ appointment or as early as possible after this. At this appointment the midwife will explain to the woman that she will receive a resource pack to help her perform PFME. Each resource pack will include an APPEAL leaflet with PFME information, a link to APPEAL developed videos, a card with links to recommended Apps to download to support PFME performance, and some promotional items (e.g. APPEAL logo stickers) to use as reminders. Women will be asked at all subsequent antenatal appointments about PFME progress (with adaptation of personalised PFME training programme where necessary) and any problems with PFME or UI symptoms.

Each intervention cluster will include a midwife ‘champion’ from the team who will receive additional training on how to support and manage women whose UI symptoms may be more severe or giving cause for concern, including appropriate referral pathways. Champions will also provide reminders and advice for midwives in their team. Midwives will be given 2 to 3 months (depending on date of their training) to practice implementing the PFME intervention into their routine care.

### Outcomes

The primary outcome is feasibility of undertaking a definitive future trial which we will assess in the following ways:

- Questionnaire return rates from women who give birth over a pre-selected 1-month period at 10–12 weeks postpartum overall and across trial arms
- Prevalence of UI at 10–12 weeks using the ICIQ-UI SF [20], and faecal incontinence at 10–12 weeks using the Revised Faecal Incontinence Scale [21]
- Women’s practice of PFME, their adherence and confidence using self-efficacy [22] and Exercise Adherence Rating Scales [23]

Secondary outcomes will

- Assess if the intervention can be implemented by midwives as part of routine antenatal care
- Assess midwife support for PFME in both trial arms from responses in women’s 10–12-week postpartum questionnaire to assess possible intervention contamination in the control clusters
- Assess women’s experiences in both trial arms of midwifery advice and support to perform PFME during pregnancy

The following progression criteria should be met to inform progression to a full RCT:

- Questionnaire return rate from women across trial arms is not likely to result in substantial bias, as indicated by either a high overall return rate and/or that women who return questionnaires in both trial arms have similar baseline characteristics
- Women’s self-reported adherence to performing PFME is higher among those in intervention clusters than in controls
- Midwife support for PFME is reported as greater by women in the intervention than the control clusters

We have decided not to quantify particular progression criteria because the decision will be made based on a combination of criteria findings. The research team would seek the advice of the Trial Steering Committee (TSC) about progression to full trial when information on the above criteria has been obtained.

### Study procedures

#### Consent process

At their first postnatal contact, women in both trial arms who give birth over a pre-selected 1-month period will be informed by their midwife that they will receive a questionnaire and cover letter (comprising a short Patient Information Sheet) at 10**–**12 weeks postpartum. As the study involves low risk for participants, this approach complies with Health Research Authority guidance, section 2.5 on ‘applying a proportionate approach to the process of seeking consent’ [24]. All women who received antenatal care in the trial clusters with be included, but Trust Research Midwives will exclude women who experienced a stillbirth or neonatal death, those whose infants were taken into care due to safeguarding concerns and women with a current severe mental health problem, providing these data are available in the woman’s maternity records.

Women’s consent for their data to be used will comprise completion and return of their questionnaire. The cover letter will clearly state that by completing the questionnaire women agree to allow their responses to be used in the study. The letter will also ask the woman for permission for a Research Midwife from the study maternity unit to obtain relevant data items including socio-demographic characteristics, labour and birth outcomes and infant birth data from their maternity notes. The letter will stress that participation is voluntary with patient identifiers only accessible to Research Midwives at the study site. The letter will also ask if women would be prepared to provide their contact details for a member of the research team to contact them to arrange a short interview about their experiences of midwifery advice and support for PFME in pregnancy.

To reach women who do not read English as a first language, letters and questionnaires will be translated into the five other most commonly spoken languages at our study sites; Urdu, Pashto, Romanian, Polish, and Arabic. As not all outcome measures have been translated and validated in these languages, a shortened version of the questionnaire will be sent in these cases.

Permission to recruit midwifery teams was provided early in the trial set-up by heads of midwifery and community midwifery matrons at each NHS site.

#### Randomisation

Midwifery community-based teams (clusters) will be randomised in a 1:1 ratio to standard care only or standard care plus intervention. A minimisation algorithm implemented in Microsoft Excel by the study statistician will be used to ensure approximate balance over the following variables:

- Midwifery team size defined by number of births (≤median monthly bookings vs >median monthly bookings)
- NHS maternity unit

A ‘random element’ will be included in the minimisation algorithm, so that each cluster has a probability of being randomised to the opposite treatment that they would have otherwise received.

Blinding of midwives providing care will not be possible due to the nature of the intervention. Women receiving antenatal care will not explicitly be blinded but the PFME support they experience will be the usual care provided by their midwives. Those responsible for conducting the trial analysis (but not the process evaluation) will be blind to allocation.

#### Participant identification

Research Midwives employed by the NHS maternity units will work through the records of women who give birth under the care of the community midwifery-teams (clusters) during the pre-defined sample month and obtain their hospital number, name and address. Each woman will be allocated a unique study number to link their de-identified data from their questionnaires and maternity records prior to being entered onto a bespoke study database hosted by the local Clinical Trials Unit.

#### Women’s questionnaires at 10–12 weeks

The questionnaire pack will include a request to return the completed questionnaire to the unit Research Midwife in a pre-paid self-addressed envelope. A £10 voucher will be given to ‘thank’ women who return completed questionnaires. Table 2 shows data collection processes and timing.

Responses from completed questionnaires will be entered by a Research Midwife employed by the NHS maternity unit. The Research Midwives will provide a list of the hospital/NHS numbers of the women who grant permission for their maternity records to be accessed to their local IT Department to obtain information on:

- Woman’s age, ethnicity, parity, onset of labour (spontaneous or induced), mode of birth (spontaneous vaginal, instrumental, caesarean section), anaesthetic/analgesia used, perineal trauma, episiotomy, and duration of active second stage.
- Baby’s gestation at birth, birthweight, and head circumference.

**Table 2:** APPEAL data collection process and timing

Outcome/data	Timing of data capture	Process
Midwifery team characteristics (clusters); midwife experience of trial processes	Various times throughout the cluster trial process	Number of births per team, number and grades of midwives per team and team location, number of women/births per team from NHS trust managers/community-team leads. Interviews with individual midwives in both study arms.
Health outcomes & PFME performance	Questionnaire follow up of all women in participating clusters at 10-12 weeks postpartum.	Overseen by local NHS trust Research Midwives. If no response to initial follow up, questionnaire resent. If no response after a further two weeks, other options will include sending a text message reminder
Data from women’s maternity records	On return of consent in women’s questionnaire.	Extracted by local trust Research Midwives from maternity records.
Data from telephone/online interviews with women from both trial arms	Interviews with 10-15 women from each trial arm	A sample of up to 30 women who agreed to be approached. Interviews to qualitatively explore their experiences of receiving (or not) information and advice about doing PFME during pregnancy; and to explore any possible contamination between arms.
Intervention cluster midwives’ views of training	Questionnaire to all midwives attending training, at start & end of the training session. On completing training roll-out in intervention clusters. Up to 15 midwives (including champions).	To evaluate the acceptability and effect on confidence of the training. Purposive sampling to represent cluster site/size. Telephone/online interviews during the practice month to assess acceptability, fidelity to intervention.
APPEAL midwives’ views of implementation of the intervention	Prior to completion of roll-out. All midwives in intervention clusters	Questionnaire to explore how midwives administered the intervention in practice, how often PFME were discussed with women at routine AN contacts, barriers and facilitators to implementation
APPEAL midwife champions views of role supporting roll-out and implementation of training intervention	On completion of intervention roll-out.	The APPEAL champions included in the aforementioned intervention cluster interviews. Telephone/online interviews to assess acceptability, fidelity to intervention pertaining to the champion role.
Midwives’ views on acceptability of the intervention/standard PFME advice (intervention and control clusters) and study processes (intervention and control midwives)	Interviews with up to 15 midwives from each trial arm.	Purposive sampling to represent cluster site/size. Telephone/online interviews to assess acceptability, fidelity to intervention (intervention) and potential contamination (control).

#### Women’s interviews

Women who agree to be approached will be purposively sampled from both trial arms based on their midwife team and location and invited to take part in one-to-one telephone or online audio interviews (using a secure web-based platform, i.e. Zoom). Up to 15 women from each arm will be interviewed.

Interviews will explore their views of doing PFME during pregnancy, whether they experienced incontinence and what support or advice they received from midwives or other health care providers. They will be asked about their current continence status, practice of PFME and if they have heard anything about the APPEAL study to enable the researchers to further estimate the level of any cross contamination from the intervention groups. Interviews will take place after the women have completed the 10**–**12-week questionnaire.

#### Participant withdrawal

A letter included with the 10–12-week questionnaire will make clear that women are under no obligation to participate. If a request to withdraw data is received after initial analysis their data will not be included in further analysis

#### Midwife pre- and post-training questionnaires

All community midwives from intervention clusters will be asked to complete a pre and post-training questionnaire immediately after their training session. They will be provided with a link during the session to complete the questionnaires online (using REDCap). Collected data will be pseudo-anonymised.

#### Midwife interviews

About 15 midwives, including APPEAL champions will be invited to take part in one-to-one interviews during the initial implementation period. Interviews will explore their views on the training and the PFME intervention for women.

Following intervention implementation and after first postnatal contact (when midwives have advised women to expect a questionnaire), approximately 15 intervention and 15 control midwives will be invited for telephone interview. Midwives will be purposively selected to reflect team size and location to explore their views on current PFME provision, how and when they advise women on PFME as well as experiences of intervention implementation and trial process for intervention midwives.

#### Midwife intervention implementation

Via APPEAL midwife ‘champions’ for each cluster, the intervention community midwives will be invited to complete a short-questionnaire on barriers and facilitators to implementation of the APPEAL intervention.

Midwives participating in the process evaluation will be informed that they do not need to participate and if they do, but subsequently change their mind, they can request their data are not included in further analysis.

#### Sample size

The sample size was based on number of women and clusters needed to estimate the return rate of questionnaires (across trial arms) to an acceptable level of precision.

In an earlier pilot study to test data collection instruments, 243 questionnaires were received within the specified time from the 777 that were sent (31.3%, 95% CI 27.1 to 35.4%) across 14 midwife teams (average cluster size 55). The estimated ICC for this return rate is 0.007 (95% CI 0.0005 to 0.094) (estimated using the loneway command in Stata). We take the upper limit of the 95% CI as a conservative estimate of the ‘true’ return rate ICC and assume a value of 0.10 is reasonable.

Using this estimated ICC the width of the 95% confidence interval for different rates (e.g. return rate of questionnaires) can be estimated for a given sample size allowing for inflation of standard errors due to clustering (using a *t*-distribution with *K*-1 degrees of freedom where *K* is the number of clusters). To set a conservative upper bound on the required sample size we determine the widest 95% confidence interval for a given sample size, which will occur for a rate of 50%.

To reflect changes since the earlier pilot study in the number of midwifery teams and women cared for by the teams at participating sites, the overall sample size target is around 1400 (17 clusters of average size 82). We will be able to estimate the 95% confidence interval for the return rate to a maximum width of 17.2%.

#### Statistical analysis

A separate Statistical Analysis Plan will be produced to provide a comprehensive description of planned statistical analyses. We provide an overall summary of the proposed analysis here. The primary comparison groups are women in clusters allocated to intervention versus women in clusters allocated to standard care. In the first instance, all statistical analyses will be based on the intention to treat principle. Data analysis will be descriptive and mainly focus on confidence interval estimation, with no hypothesis testing. The assessment of missing data is an outcome measure of this feasibility and pilot trial.

We will report numbers contributing and numbers missing for all variables and descriptive statistics for all outcomes. Analysis methods will be chosen according to the data type of the outcome under investigation, in brief:

- Continuous endpoints (e.g. confidence scores elicited on a range 0**–**32): These data will be summarised using means and standard deviations, by arm
- Categorical (dichotomous) endpoints (e.g. experience of UI in the past 4 weeks): The number of participants and percentages experiencing the event will be summarised by arm

For all total scores and dichotomous feasibility outcomes (e.g. proportion of women returning questionnaires) summary measures and 95% confidence intervals per trial arm will be estimated using a cluster level analysis, the *t*-distribution with df = *K*-1 and appropriate transformation where necessary (and weighting if there is variation in cluster sizes). This approach will appropriately allow for the clustered nature of the trial.

Quantitative data from intervention cluster midwives pre- and post-training session and intervention implementation questionnaires will be summarised using descriptive statistics and where appropriate inferential analysis undertaken using repeated measures statistical tests.

The final dataset will be available to members of the Trial Management and co-applicant group who need access to the data to undertake the final analyses.

Requests for access to data from the APPEAL trial should be addressed to APPEAL@trials.bham.ac.uk. Anonymised individual participant data collected during the trial will be available with no end date. All proposals requesting data access will need to specify how the data will be used, and will need the approval of the trial management group prior to data release.

#### Qualitative data

Adherence to implementing the PFME training and fidelity to the intervention will be described through qualitative methods using interviews with midwives who provided care in intervention clusters. Women’s views of support for performing PFME in pregnancy will also be described through qualitative methods using interviews.

Qualitative data in free text responses from intervention cluster midwives pre- and post-training session and intervention implementation questionnaires will be analysed using content analysis.

The interview audio data from midwives and women will be transcribed verbatim. The transcripts will be checked with initial familiarisation process by one researcher who will begin the coding process. A second researcher will independently code a subset of transcripts before coming together to discuss and agree the final coding framework. The principles of the framework method will be used for this process. For each qualitative data set, emerging themes will be charted and data from transcripts summarised under each theme.

A mixed-methods approach will integrate results from each data source [25] affording a fuller picture of the results with ‘triangulation’ occurring at the interpretation stage after each data source has been analysed separately. A summary matrix will be created with an assessment of whether there is agreement, partial agreement, disagreement or ‘silence’ (no data informing that theme) from the differing data sources.

#### Patient and public involvement

Patient and public involvement (PPI) has occurred throughout all phases of APPEAL programme: from developing the original study questions for each of the four Work Packages (including the protocol for the feasibility and pilot cluster trial reported here) to APPEAL logo design.

For the feasibility and pilot cluster trial reported here, the PPI group contributed to the design and choice of resources midwives would provide to women (leaflet, APP card, reminder sticker with APPEAL logo, bag) to remind them to perform their PFME during pregnancy, and how to perform PFME.

Our PPI representatives checked and commented on the participant information sheet and topic guide for the women’s interviews. The PPI group will meet to discuss preliminary trial and process evaluation findings and their views/discussion points used to inform interpretation dissemination of findings.

#### Dissemination

A lay summary of the study will be available on the National Institute for Health Research website. Final results will be publicly available through open-access publication in a peer-reviewed journal and presented at relevant conferences and research meetings. The PPI group will contribute to the dissemination plan.

## Discussion

Urinary incontinence is a widespread and persistent problem following birth, with good evidence that PFME in pregnancy can prevent it, yet few women are advised to perform PFME in pregnancy, and training to enable midwives to support women to manage this aspect of their health at antenatal contacts is not provided. This feasibility and pilot cluster trial was designed to provide evidence of whether a definitive trial of effectiveness could be undertaken.

The trial team will also have to consider other external factors. Following a trial pause due to COVID-19 it became apparent that a future definitive cluster trial of effectiveness may not be possible. This is due to changes to routine care starting to be implemented as part of the NHS England Long-Term Plan to improve women’s pelvic floor health, which will include training of midwives to support women to undertake PFME in pregnancy as well as postnatal access to physiotherapy and multi-disciplinary pelvic health clinics by 2023/2024. NHS England (NHSE) had been expecting APPEAL feasibility and pilot trial findings to inform this plan but early implementation funding provided by NHSE could not be paused. It is expected that new services will be in place across England by 2024. It may therefore be that a full RCT, as was our original aim, will not be possible. However, findings of the APPEAL feasibility and pilot trial will inform and support the NHSE ambition for better perinatal pelvic floor health and will inform services elsewhere internationally.

## Data Availability

Data sharing is not applicable to this article as no datasets were generated or analysed during the current study
